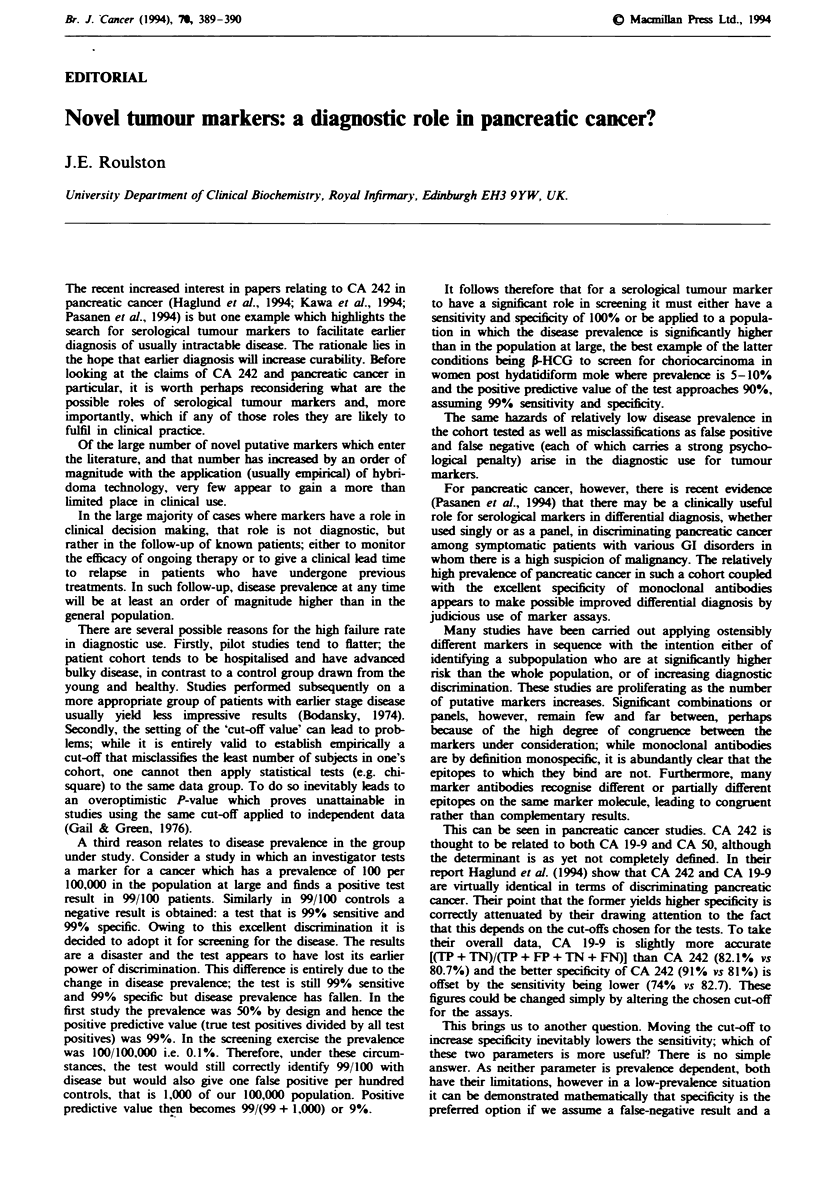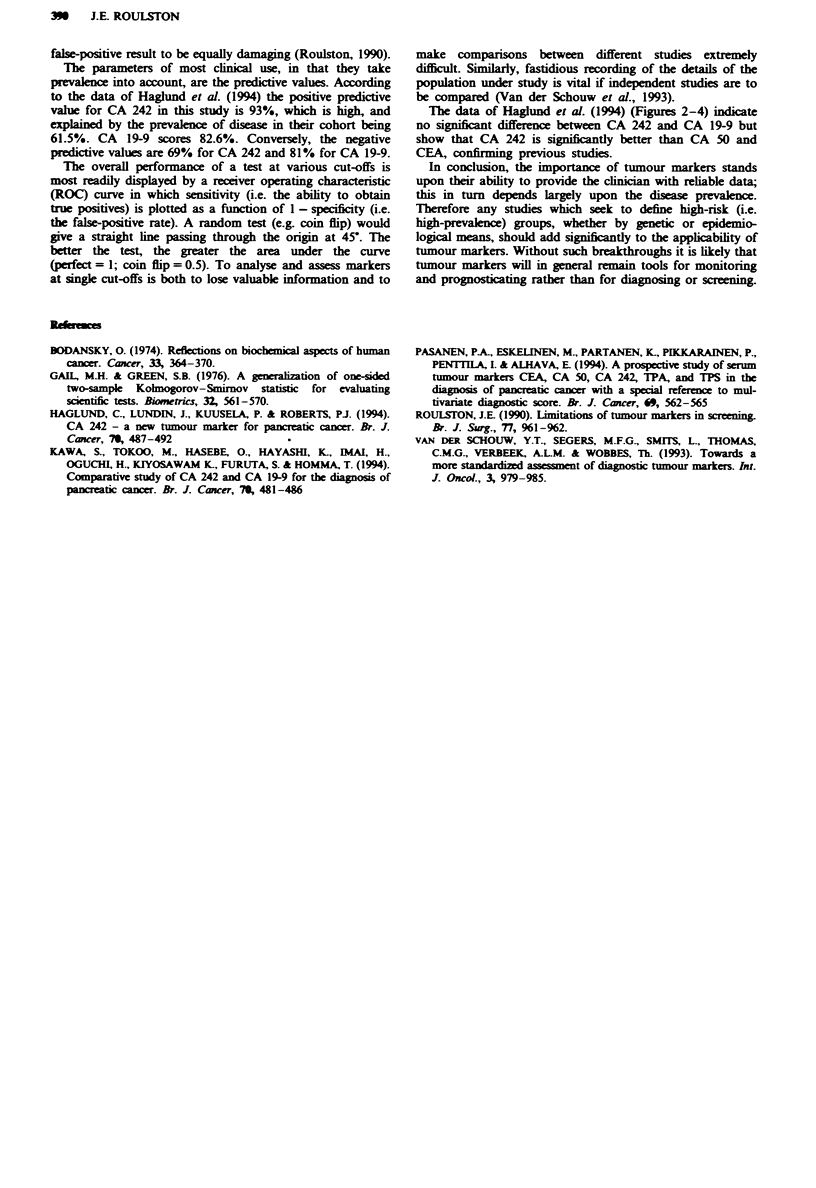# Novel tumour markers: a diagnostic role in pancreatic cancer?

**DOI:** 10.1038/bjc.1994.313

**Published:** 1994-09

**Authors:** J. E. Roulston


Br. J. Cancer (1994), 70, 389-390 © Macmillan Press Ltd., 1994

EDITORIAL

### Novel tumour markers: a diagnostic role in pancreatic cancer?

J.E. Roulston

*University Department of Clinical Biochemistry, Royal Infirmary, Edinburgh EH3 9YW, UK.*

The recent increased interest in papers relating to CA 242 in pancreatic cancer (Haglund *et al.*, 1994; Kawa *et al.*, 1994; Pasanen *et al.*, 1994) is but one example which highlights the search for serological tumour markers to facilitate earlier diagnosis of usually intractable disease. The rationale lies in the hope that earlier diagnosis will increase curability. Before looking at the claims of CA 242 and pancreatic cancer in particular, it is worth perhaps reconsidering what are the possible roles of serological tumour markers and, more importantly, which if any of those roles they are likely to fulfil in clinical practice.

Of the large number of novel putative markers which enter the literature, and that number has increased by an order of magnitude with the application (usually empirical) of hybridoma technology, very few appear to gain a more than limited place in clinical use.

In the large majority of cases where markers have a role in clinical decision making, that role is not diagnostic, but rather in the follow-up of known patients; either to monitor the efficacy of ongoing therapy or to give a clinical lead time to relapse in patients who have undergone previous treatments. In such follow-up, disease prevalence at any time will be at least an order of magnitude higher than in the general population.

There are several possible reasons for the high failure rate in diagnostic use. Firstly, pilot studies tend to flatter; the patient cohort tends to be hospitalised and have advanced bulky disease, in contrast to a control group drawn from the young and healthy. Studies performed subsequently on a more appropriate group of patients with earlier stage disease usually yield less impressive results (Bodansky, 1974). Secondly, the setting of the 'cut-off value' can lead to problems; while it is entirely valid to establish empirically a cut-off that misclassifies the least number of subjects in one's cohort, one cannot then apply statistical tests (e.g. chi-square) to the same data group. To do so inevitably leads to an overoptimistic *P*-value which proves unattainable in studies using the same cut-off applied to independent data (Gail & Green, 1976).

A third reason relates to disease prevalence in the group under study. Consider a study in which an investigator tests a marker for a cancer which has a prevalence of 100 per 100,000 in the population at large and finds a positive test result in 99/100 patients. Similarly in 99/100 controls a negative result is obtained: a test that is 99% sensitive and 99% specific. Owing to this excellent discrimination it is decided to adopt it for screening for the disease. The results are a disaster and the test appears to have lost its earlier power of discrimination. This difference is entirely due to the change in disease prevalence; the test is still 99% sensitive and 99% specific but disease prevalence has fallen. In the first study the prevalence was 50% by design and hence the positive predictive value (true test positives divided by all test positives) was 99%. In the screening exercise the prevalence was 100/100,000 i.e. 0.1%. Therefore, under these circumstances, the test would still correctly identify 99/100 with disease but would also give one false positive per hundred controls, that is 1,000 of our 100,000 population. Positive predictive value then becomes 99/(99 + 1,000) or 9%.

It follows therefore that for a serological tumour marker to have a significant role in screening it must either have a sensitivity and specificity of 100% or be applied to a population in which the disease prevalence is significantly higher than in the population at large, the best example of the latter conditions being β-HCG to screen for choriocarcinoma in women post hydatidiform mole where prevalence is 5-10% and the positive predictive value of the test approaches 90%, assuming 99% sensitivity and specificity.

The same hazards of relatively low disease prevalence in the cohort tested as well as misclassifications as false positive and false negative (each of which carries a strong psychological penalty) arise in the diagnostic use for tumour markers.

For pancreatic cancer, however, there is recent evidence (Pasanen *et al.*, 1994) that there may be a clinically useful role for serological markers in differential diagnosis, whether used singly or as a panel, in discriminating pancreatic cancer among symptomatic patients with various GI disorders in whom there is a high suspicion of malignancy. The relatively high prevalence of pancreatic cancer in such a cohort coupled with the excellent specificity of monoclonal antibodies appears to make possible improved differential diagnosis by judicious use of marker assays.

Many studies have been carried out applying ostensibly different markers in sequence with the intention either of identifying a subpopulation who are at significantly higher risk than the whole population, or of increasing diagnostic discrimination. These studies are proliferating as the number of putative markers increases. Significant combinations or panels, however, remain few and far between, perhaps because of the high degree of congruence between the markers under consideration; while monoclonal antibodies are by definition monospecific, it is abundantly clear that the epitopes to which they bind are not. Furthermore, many marker antibodies recognise different or partially different epitopes on the same marker molecule, leading to congruent rather than complementary results.

This can be seen in pancreatic cancer studies. CA 242 is thought to be related to both CA 19-9 and CA 50, although the determinant is as yet not completely defined. In their report Haglund *et al.* (1994) show that CA 242 and CA 19-9 are virtually identical in terms of discriminating pancreatic cancer. Their point that the former yields higher specificity is correctly attenuated by their drawing attention to the fact that this depends on the cut-offs chosen for the tests. To take their overall data, CA 19-9 is slightly more accurate [(TP + TN)/(TP + FP + TN + FN)] than CA 242 (82.1% *vs* 80.7%) and the better specificity of CA 242 (91% *vs* 81%) is offset by the sensitivity being lower (74% *vs* 82.7). These figures could be changed simply by altering the chosen cut-off for the assays.

This brings us to another question. Moving the cut-off to increase specificity inevitably lowers the sensitivity; which of these two parameters is more useful? There is no simple answer. As neither parameter is prevalence dependent, both have their limitations, however in a low-prevalence situation it can be demonstrated mathematically that specificity is the preferred option if we assume a false-negative result and a false-positive result to be equally damaging (Roulston, 1990).

The parameters of most clinical use, in that they take prevalence into account, are the predictive values. According to the data of Haglund *et al.* (1994) the positive predictive value for CA 242 in this study is 93%, which is high, and explained by the prevalence of disease in their cohort being 61.5%. CA 19-9 scores 82.6%. Conversely, the negative predictive values are 69% for CA 242 and 81% for CA 19-9.

The overall performance of a test at various cut-offs is most readily displayed by a receiver operating characteristic (ROC) curve in which sensitivity (i.e. the ability to obtain true positives) is plotted as a function of 1 − specificity (i.e. the false-positive rate). A random test (e.g. coin flip) would give a straight line passing through the origin at 45°. The better the test, the greater the area under the curve (perfect = 1; coin flip = 0.5). To analyse and assess markers at single cut-offs is both to lose valuable information and to make comparisons between different studies extremely difficult. Similarly, fastidious recording of the details of the population under study is vital if independent studies are to be compared (Van der Schouw *et al.*, 1993).

The data of Haglund *et al.* (1994) (Figures 2–4) indicate no significant difference between CA 242 and CA 19-9 but show that CA 242 is significantly better than CA 50 and CEA, confirming previous studies.

In conclusion, the importance of tumour markers stands upon their ability to provide the clinician with reliable data; this in turn depends largely upon the disease prevalence. Therefore any studies which seek to define high-risk (i.e. high-prevalence) groups, whether by genetic or epidemiological means, should add significantly to the applicability of tumour markers. Without such breakthroughs it is likely that tumour markers will in general remain tools for monitoring and prognosticating rather than for diagnosing or screening.

### References

BODANSKY, O. (1974). Reflections on biochemical aspects of human cancer. *Cancer*, **33**, 364–370.

GAIL, M.H. & GREEN, S.B. (1976). A generalization of one-sided two-sample Kolmogorov–Smirnov statistic for evaluating scientific tests. *Biometrics*, **32**, 561–570.

HAGLUND, C., LUNDIN, J., KUUSELA, P. & ROBERTS, P.J. (1994). CA 242 – a new tumour marker for pancreatic cancer. *Br. J. Cancer*, **70**, 487–492

KAWA, S., TOKOO, M., HASEBE, O., HAYASHI, K., IMAI, H., OGUCHI, H., KIYOSAWAM K., FURUTA, S. & HOMMA, T. (1994). Comparative study of CA 242 and CA 19-9 for the diagnosis of pancreatic cancer. *Br. J. Cancer*, **70**, 481–486

PASANEN, P.A., ESKELINEN, M., PARTANEN, K., PIKKARAINEN, P., PENTTILA, I. & ALHAVA, E. (1994). A prospective study of serum tumour markers CEA, CA 50, CA 242, TPA, and TPS in the diagnosis of pancreatic cancer with a special reference to multivariate diagnostic score. *Br. J. Cancer*, **69**, 562–565

ROULSTON, J.E. (1990). Limitations of tumour markers in screening. *Br. J. Surg.*, **77**, 961–962.

VAN DER SCHOUW, Y.T., SEGERS, M.F.G., SMITS, L., THOMAS, C.M.G., VERBEEK, A.L.M. & WOBBES, Th. (1993). Towards a more standardized assessment of diagnostic tumour markers. *Int. J. Oncol.*, **3**, 979–985.